# Oocyte Morphometric Assessment and Gene Expression Profiling of Oocytes and Cumulus Cells as Biomarkers of Oocyte Competence in Sheep

**DOI:** 10.3390/ani11102818

**Published:** 2021-09-27

**Authors:** Carolina Maside, Irene Sánchez-Ajofrín, Daniela Medina-Chávez, Benner Alves, José Julián Garde, Ana Josefa Soler

**Affiliations:** 1 SaBio IREC (CSIC-UCLM-JCCM), ETSIAM, Campus Universitario, s/n, 02071 Albacete, Spain; irene.ssanchez@uclm.es (I.S.-A.); daniela.medina@uclm.es (D.M.-C.); julian.garde@uclm.es (J.J.G.); anajosefa.soler@uclm.es (A.J.S.); 2 Biotechnology of Animal and Human Reproduction (TechnoSperm), Institute of Food and Agricultural Technology, University of Girona, 17003 Girona, Spain; 3 Unit of Cell Biology, Department of Biology, Faculty of Sciences, University of Girona, 17003 Girona, Spain; 4 Ovid Research Company, Berkeley, CA 94710, USA; bennervet@gmail.com

**Keywords:** biomarkers, oocyte quality, sheep

## Abstract

**Simple Summary:**

In vitro production (IVP) of embryos is an essential element of many reproductive biotechnologies. Although some remarkable achievements have been obtained for ovine IVP, the overall efficiency is still low. A limiting factor of the technique is the great variability of the oocyte quality used in IVP. The current selection criterion of oocytes is based on a morphological factor, which is insufficient to determine oocytes’ competence. Therefore, the identification of biomarkers for the selection of oocytes of high developmental competence is a major research goal. The purpose of this study was to evaluate the morphometric parameters of oocytes and the relative gene expression of oocytes and cumulus cells (CCs) as biomarkers of oocyte quality after individually culturing them in order to increase the final efficiency of the IVP technique in sheep. We observed that higher blastocyst rates were obtained from oocytes classified as intermediate and large according to total diameter, oocyte diameter, and zona pellucida thickness. Moreover, the expression of genes related to oocyte quality was higher in oocytes classified as large and in CCs from oocytes classified as large and thus able to reach the blastocyst stage. Oocyte morphometric assessments and gene expression in CCs may be used as biomarkers of oocyte quality.

**Abstract:**

Oocyte quality is crucial for subsequent embryo development and so it is a major challenge in assisted reproductive technologies. The aim of the present work was to evaluate the morphometric parameters of oocytes (experiment 1) and the relative gene expression of oocytes and cumulus cells (CCs) (experiment 2) as biomarkers of oocyte quality after individually culturing them (one oocyte or embryo/drop). In experiment 1, individually matured oocytes were measured and classified into small, intermediate, and large oocytes after a cluster analysis, based on total diameter (with zona pellucida, ZP), oocyte diameter (without ZP), and ZP thickness. These oocytes were individually fertilized in vitro and cultured. The embryo development was evaluated up to the blastocyst stage. According to the total diameter, oocyte diameter, and ZP thickness, the blastocyst rate decreased in the small oocytes group (3.1 ± 3.1, 14.1 ± 9.4, and 26.7 ± 3.9, respectively) compared to the intermediate (29.4 ± 5.2, 30.5 ± 10.1, and 28.6 ± 9.6, respectively) and large oocytes groups (54.2 ± 13.5, 44.4 ± 3.9, and 67.6 ± 12.4, respectively). In addition, the probability of reaching the blastocyst stage was positively related to the total diameter (*p* < 0.001), oocyte diameter (*p* < 0.05), and ZP thickness (*p* < 0.001). Furthermore, the relative gene expression of *BAX*, *BCL2*, *GDF9*, and *GJA1* was lower in oocytes classified as large. In experiment 2, the mRNA transcript relative abundance pattern of genes in CCs was evaluated according to oocyte total diameter and developmental stage reached. CCs from oocytes classified as large and oocytes capable of developing to the blastocyst stage had a lower relative expression of *BAX*, *STAR*, and *PTGS2*, while a higher expression of *HAS2* and *SDC2* transcript was observed for those oocytes. In conclusion, oocyte morphometric parameters and gene expression analysis in oocytes and CCs provide methods for the identification of the most competent oocytes for assisted reproductive technologies in sheep.

## 1. Introduction

In vitro production (IVP) of embryos is an essential instrument for many reproductive biotechnologies such as nuclear transfer, cloning, gene editing, and stem cell research. Moreover, IVP in sheep is of great interest to accelerate breeding and improve the productivity and profitability of livestock. Despite some remarkable achievements obtained for the IVP of ovine embryos, its overall efficiency is still low, with only 20–50% of oocytes reaching the blastocyst stage [1]. One of the main reasons for the low blastocyst rates is the high variability in the quality of oocytes used for in vitro maturation (IVM). Oocyte quality is crucial for subsequent embryo development [2,3,4,5]. Developmental competence is acquired during folliculogenesis as the oocyte grows and during oocyte maturation (from the primordial follicle stage to ovulation) while acquiring the cytoplasmic organelles, proteins, and RNAs required for postfertilization development [5,6]. The IVP technique includes three successive procedures: IVM, a crucial step to provide high-quality oocytes, in vitro fertilization (IVF), and in vitro culture (IVC) of embryos. Oocytes selected for IVM are retrieved from follicles at different stages of folliculogenesis; most, although meiotically competent, have not undertaken all essential cytoplasmic changes to support further development. Moreover, the selection criteria currently used are based on subjective morphological parameters such as the homogeneity of the cytoplasm or the number of cumulus cell layers, and these may be insufficient to accurately differentiate oocytes of high or low quality [6,7,8,9]. Therefore, it is not surprising that methods for the selection of oocytes with the best developmental potential have been the focus of intense research for the last few decades. Previous studies in cattle and goats have shown that the size of oocytes is correlated with competence and development [10,11,12,13]. In bovine and caprine livestock, a minimum oocyte diameter of 115 μm is necessary for full meiotic competence, and full developmental competence is acquired at 120 μm [12,14,15].

During folliculogenesis, the oocyte is surrounded by follicular cells that differentiate at the antral stage into mural granulosa cells and cumulus cells (CCs), establishing a bidirectional communication between the oocyte and surrounding CCs. This communication is critical for follicular compartment development, oocyte maturation, and competence acquisition [16,17,18]. During IVP procedures, CCs are separated from the oocytes and discarded, making them a source of material for analysis with the aim of defining the competence of oocytes. Several studies in humans suggest that CCs may be used to gain insight into the viability and reproductive potential of the oocytes [19,20]. Moreover, previous studies showed that the relative mRNA expression of gremlin 1 (*GREM1*), hyaluronan synthase 2 (*HAS2*), prostaglandin-endoperoxide synthase 2 (*PTGS2*), growth differentiation factor 9 (*GDF*-9), pentraxin 3 (*PTX3*), epidermal growth factor receptor (EGFR), and steroidogenic acute regulatory protein (*STAR*) in CCs was related to oocyte development capacity and embryo morphology [20,21]. Furthermore, the mRNA transcript relative abundance patterns in CCs were identified as possible markers of bovine oocyte competence [22] or blastocyst formation [23].

With this background, the aim of the work was to evaluate whether the morphometric parameters of oocytes and the relative genes expression of oocytes and CCs subjected to individual IVP systems could be used as possible biomarkers of oocyte quality in IVP embryos.

## 2. Materials and Methods

### 2.1. Chemicals, Culture Media and Culture Conditions

Unless otherwise indicated, all chemicals used in the present study were acquired from Merck Life Sciences (Madrid, Spain). The ovaries were transported in 0.9% (*w*/*v*) NaCl supplemented with 0.1% gr/L penicillin and 0.1% gr/L streptomycin. Cumulus–oocyte complexes (COCs) were recovered in tissue culture medium 199 (TCM199) supplemented with 2.38 mg/mL N-(2-Hydroxyethyl)piperazine-N′-(2-ethanesulfonic acid), 4-(2-Hydroxyethyl)piperazine-1-ethanesulfonic acid (HEPES), 2 μL/mL heparin, and 4 μL/mL gentamicin (collected medium), and then washed in TCM199 supplemented with 4 μL/mL gentamycin (washing medium). The maturation medium used was TCM199 supplemented with 4 μL/mL gentamycin, 100 μM cysteamine, 10 mg/mL follicle-stimulating hormone, 10 mg/mL luteinizing hormone, and 10% fotal calf serum [24]. The medium used for in vitro fertilization was synthetic oviductal fluid (SOF) enriched with 10% estrous sheep serum [24]. A SOF media supplemented with 2.35 mg/mL HEPES (SOF–HEPES) was used for washing spermatozoa after thawing and before capacitation. The embryo culture medium used was SOF, supplemented with 3 mg/mL of bovine serum albumin (SOF–BSA), as described by Takahashi and First [25]. In vitro maturation and fertilization were performed under mineral oil at 38.5 °C in 5% CO_2_ in air and 98% relative humidity. In vitro embryo culture was performed under mineral oil at 38.5 °C in 5% CO_2_, 5% O_2_, and 90% N2 in air with high humidity.

### 2.2. Experimental Design

Oocytes (*n* = 199) were submitted to individual IVM, IVF, and IVC (one oocyte or embryo per drop). Routine IVM, IVF, and IVC protocols were performed where oocytes (*n* = 167) were cultured in groups (45–50 COCs or embryos per drop) as a control for the IVP procedure. In experiment 1, after IVM, images of individual matured oocytes (*n* = 133) were captured for later measurement. The measurements were: total diameter (with zona pellucida–ZP), oocyte diameter (without ZP), and ZP thickness. Then, individual IVF and IVC were performed and evaluated for each oocyte development to obtain the following classifications: (i) undeveloped: oocytes that were unable to be fertilized or to develop to the 2–8-cell stage; (ii) total embryos at 2–8-cell stage: all oocytes that were able to develop to the 2–8-cell stage; (iii) embryos arrested at the 2–8-cell stage: oocytes that reached the 2–8-cell stage but were unable to develop into blastocysts; (iv) blastocysts: oocytes that were able to develop to the blastocyst stage. In addition, gene expression was analyzed in oocytes (*n* = 45), classified according to the total diameter of oocyte. In experiment 2, the relative gene expression levels of CCs from COCs (*n* = 117) matured in vitro individually. Cumulus cells were classified according to the total diameter (*n* = 45) and developmental stage reached (*n* = 72) (described above) by the native oocyte. All experiments were conducted four times. 

### 2.3. Oocyte Collection and In Vitro Maturation (IVM)

Ovaries were obtained from adult ewes around six years old (from January to May) at a slaughterhouse transported to the laboratory at 30 °C. Cumulus–oocyte complexes were recovered by slicing the ovaries with scalpels and placing in collected TCM199 medium. Only those COCs whose oocytes were surrounded by a complete, compact cumulus mass and that had evenly granulated cytoplasm were selected and washed in a washing medium. Then, COCs were randomly distributed in two groups: (i) group IVM (control): groups of 45–50 COCs were placed into each well of a four-well multidish (NUNC) containing 500 µL of pre-equilibrated maturation media; (ii) individual IVM: individual COCs were placed in 10 µL drops of IVM medium (1 COC/drop). The COCs were then incubated for 24 h. One part of the in vitro matured oocytes (10–15 oocytes for replicate) was analyzed to determine maturation rates. 

### 2.4. In Vitro Fertilization (IVF)

After maturation, the COCs were partially denuded by gentle pipetting. Cumulus cells obtained from individual COCs were washed in PBS-BSA and stored at –80 °C until use. Denuded oocytes were divided into: (i) group IVF (control): groups of 40–45 oocytes were placed into four-well plates containing 450 μL of fertilization medium; (ii) individual IVF: oocytes were placed individually in 25 µL drops of fertilization medium (1 oocyte/drop). Frozen–thawed ram semen was centrifuged on a 45/90 discontinuous Percoll gradient for 10 min at 2900 rpm. The pellet was resuspended in 0.5 mL SOF–HEPES and washed for 5 min at 700 rpm. The final pellet concentration was determined using a Bürker chamber and adjusted to a final concentration at 10 × 10^6^ spermatozoa per milliliter. Sperm was capacitated for 15 min at 38 °C in 5% CO_2_ with fertilization media. Oocytes and sperm were co-incubated at a final concentration of 10^6^ spermatozoa per millilitre for 18 h at 38.5 °C in 5% CO_2_. One part of the in vitro fertilized oocytes (10–15 presumptive zygotes for replicate) was analyzed to determine the fertilization efficiency at 24 h postinsemination (hpi).

### 2.5. In Vitro Embryo Culture (IVC)

Twenty-four hours postinsemination, presumptive zygotes were washed of attached sperm cells and divided into: (i) group IVC (control): groups of 25 presumptive zygotes were placed into four-well plates containing 25 μL of culture medium (25 presumptive zygotes/drop); (ii) individual IVC (30 oocytes for replicate): individual presumptive zygotes were placed in 10 µL drops of culture medium (1 presumptive zygotes/drop). All zygotes were cultured for eight days. The cleavage rate was assessed at approximately 48 hpi and the blastocyst yield was recorded on days 6, 7, and 8.

### 2.6. Assessment of Maturation, Fertilization Efficiency, and Blastocyst Quality

To evaluate maturation rates, fertilization efficiency, and blastocyst quality, mature oocytes, presumptive zygotes, and expanded blastocysts were fixed in 0.5% glutaraldehyde in PBS for 20 min at room temperature and placed on a glass slide. After the addition of 1 µL SlowFace and 10 µg/mL Hoechst 33342, they were covered with a coverslip. The samples were analyzed with 20× augmentation by fluorescence microscopy (Eclipse 80i, Nikon Instruments Europe, Amsterdam, the Netherlands) using an excitation filter of 330–380 nm wavelength. The oocytes were considered mature when their chromosomes appeared organized at metaphase and showed an extruded first polar body (maturation rates). The fertilization efficiency was the number of monospermic zygotes (oocytes containing only one male pronucleus) divided by the total oocytes inseminated. Blastocyst quality was observed by nuclei cell counting of expanded blastocysts on days 6, 7, and 8.

### 2.7. Measure the Oocyte Morphometrics

After IVM, COCs were denuded by pipetting, washed, placed in SOF–BSA, and then individually measured to determine the total diameter, oocyte diameter (without ZP), and ZP thickness with a microscope equipped with a Nikon Camera and precalibrated software (ImageJ, National Institutes of Health, Bethesda, MA, USA). The values recorded were the mean of two measurements made perpendicular to each other.

### 2.8. Real-Time Quantitative PCR Analysis

After individual IVM, COCs were mechanically denuded by gentle pipetting. Denuded oocytes and CCs for gene expression analyses were collected and stored at −80 °C until being used for the PCR analysis. For RNA isolation, three pools of five oocytes classified according to the total diameter of oocytes (Experiment 1) and individual samples of CCs classified according to the total diameter and developmental stage reached by the native oocytes (Experiment 2) were collected for each experimental group. Total RNA from oocytes and CCs was isolated with a Trizol Plus Purification kit (Invitrogen, Barcelona, Spain). The RNA preparations were treated with RNeasy Micro Kit (Qiagen, Barcelona, Spain). After extraction, complementary DNA was synthesized from the isolated RNA using iScript cDNA Synthesis Kit (Bio-Rad Laboratories, Barcelona, Spain) according to the manufacturer’s protocol. The qPCR reaction was performed in a final volume of 20 µL, containing 2 µL of each complementary DNA, 1× Power SYBR Green PCR Master Mix (10 µL) (PE Applied Biosystems, Foster City, CA, USA), 6 µL of ultrapure water, and 0.5 mM of both sense and antisense primers. The gene-specific primers used for the amplification of different transcripts are shown in Table 1. The relative mRNA levels of apoptosis (*BAX*; *BCL2*), oocyte maturation (bone morphogenetic protein 15- *BMP15*; *GDF9*), growth factor (insulin-like factor receptor 2-IGF2R), and GAP junction genes (gap junction protein alpha 1-*GJA1*) were evaluated in oocytes and apoptosis (*BAX*, *BCL2*) and the acquisition of oocyte competence (*STAR*, *HAS2*, *PTGS2* and syndecan 2—*SDC2*) genes was measured in CCs. Primer specificity and amplification efficiency were verified for each gene. The real-time PCR cycling conditions consisted of 50 °C for 2 min for UDG activation, 95 °C for 2 min followed by 40 cycles of 95 °C for 15 s, and 60 °C for 1 min. All amplifications were carried out in a LightCycler 480 II (Roche, Barcelona, Spain). Two candidate reference genes, glyceraldehyde-3-phosphate-dehydrogenaseand peptidylprolyl Isomerase A (*PPIA*) and glyceraldehyde-3-phosphate-dehydrogenase (*GAPDH*), were selected as endogenous controls to study the expression, stability, and normalization of gene expression in all samples. The expression stability of these genes was analyzed using BestKeeper software. BestKeeper highlighted *PPIA* as the reference gene with the least overall variation. Two replicates were carried out for all genes and transcript levels in oocytes and CCs were normalized to the content of *PPIA*. The delta-CT method was used to transform threshold cycle values into normalized relative expression levels [26].

### 2.9. Statistical Analysis

Statistical analysis was performed using the IBM SPSS 24.0 Statistics package (SPSS, Chicago, IL, USA). The mean ± SEM of binary variables (maturation, IVF efficiency, embryos with 2–8 cells, and blastocyst rates) was obtained by calculating the variable percentage in each replicate. All variables (maturation rates, fertilization efficiency, blastocyst rate, number of cells/blastocysts, and relative gene expression) were analyzed to evaluate normality by the Kolmogorov–Smirnov test and using a mixed-model ANOVA followed by the Bonferroni post hoc test. In vitro mature oocytes were clustered by total oocyte diameter, oocyte diameter, and thickness of ZP using iterative k-means cluster analysis to classify the oocytes of the dataset into a reduced number of subpopulations according to their oocyte dimensions. The effect of clusters, as fixed variable, on developmental stage (undeveloped, total embryos at the 2–8-cell stage, embryos arrested at the 2–8-cell stage, and blastocyst rates) were calculated and compared using a mixed-model ANOVA followed by a Bonferroni post hoc test. A logistic regression was performed to analyze the relationship between morphometric parameters (total oocyte diameter, oocyte diameter, and thickness of ZP) and blastocyst formation. Differences were considered significant when *p* < 0.05.

## 3. Results

Individual IVM, IVF, and IVC did not affect the developmental competence of oocytes (Table 2). There were no differences in the percentage of maturation, IVF efficiency, cleavage, and blastocysts as well as number of cells/blastocysts between individuals and groups (control). 

### 3.1. Experiment 1: Assessment of Oocyte Morphometric Parameters as a Biomarker of Oocyte Competence

After the cluster analysis of oocyte morphometric parameters (total diameter, oocyte diameter, and ZP thickness), three groups of oocytes were clearly identified: (i) cluster 1 or small, (ii) cluster 2 or intermediate, (iii) cluster 3 or large (Table 3). 

There were significant differences in the distribution of these three subpopulations between different developmental stages by total oocyte diameter (Table 4). The percentage of undeveloped oocytes increased in small oocytes as compared with intermediate and large oocytes (*p* < 0.05). Moreover, the total number of embryos at the 2–8-cell stage and blastocyst rates were decreased in the small oocytes group compared with the intermediate and large groups (*p* < 0.05). 

Likewise, after classification by oocyte diameter (Table 5), the undeveloped rate was lower in intermediate and large oocytes than in small oocytes (*p* < 0.05). However, the intermediate and large oocytes obtained higher production rates (total number of embryos at the 2–8-cell stage and blastocyst rates) than small oocytes (*p* < 0.05).

In the same way, oocytes with small ZP thickness recorded a higher percentage of undeveloped oocytes compared to intermediate and large groups (*p* < 0.05). Moreover, the total number of embryos at the 2–8-cell stage decreased in the small oocytes group compared to the intermediate and large groups (*p* < 0.05). In addition, the large group recorded a higher blastocyst percentage than the other groups (*p* < 0.05) (Table 6).

**Figure 1:**
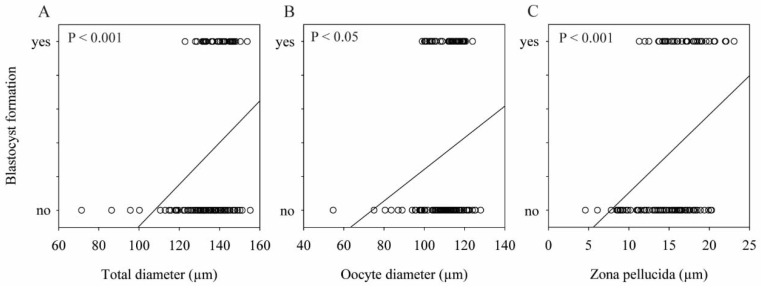
Relationship between blastocyst formation and morphometric measures: (**A**) total diameter, (**B**) oocyte diameter, and (**C**) zona pellucida thickness. Each circle on the graph represents an oocyte that was evaluated. The oocytes analyzed were defined by binary values (0 = no; 1 = yes). A logistic regression is represented by the following equations: Logit P = –13,462 + (0.0935 × total diameter), *p* < 0.001; Logit P = –6562 + (0.0530 × oocyte diameter), *p* < 0.05; Logit P = –4577 + (0.252 × total diameter), *p* < 0.001.

Based on the data from individual IVP, we observed by linear regression that the probability of having a blastocyst was positively related to the total diameter (*p* < 0.001), oocyte diameter (*p* < 0.05), and ZP thickness (*p* < 0.001) (Figure 1).

**Figure 2:**
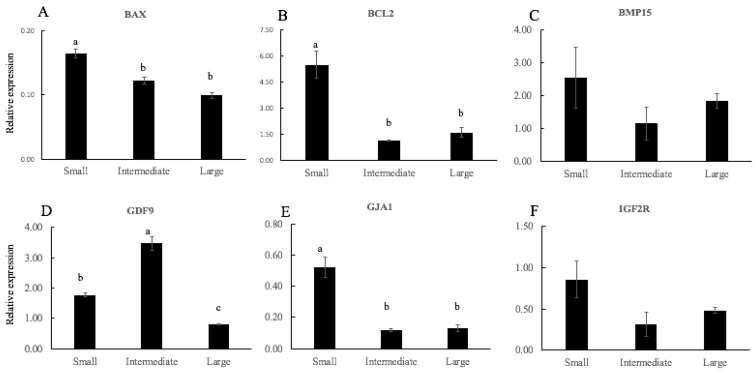
Relative gene expression of mRNA of *BAX* (**A**), *BCL2* (**B**), *BMP15* (**C**), *GDF9* (**D**), *GJA3* (**E**), and *IGF2R* (**F**) in ovine oocytes according to the clustering of total diameter (small, intermediate, or large). Different letters denote significant differences (*p* < 0.05, at least).

The relative mRNA levels of apoptosis (*BAX*, *BCL2*), oocyte maturation (*BMP15*, *GDF9*), growth factor (*IGF2R*) and GAP junction genes (*GJA1*) were quantified in oocytes classified according to cluster analysis by total diameter (small, intermediate, or large) (Figure 2). After individual IVM, BAX (Figure 2A) and BCL2 (Figure 2B) was significantly less expressed in intermediate and large oocytes than in small oocytes (*p* < 0.05). In the same way, the mRNA expression of *GJA1*(Figure 2E) was lower in small oocytes as compared to intermediate and large oocytes (*p* < 0.02). In large oocytes, *GDF9* (Figure 2D) was expressed significantly less than in small and intermediate oocytes (*p* < 0.02). However, the mRNA levels for *BMP15* (Figure 2C) and *IGFR2* (Figure 2F) did not differ between treatments. 

### 3.2. Experiment 2: Evaluation of Gene Expression of CCs as Biomarker of Oocyte Competence

**Figure 3:**
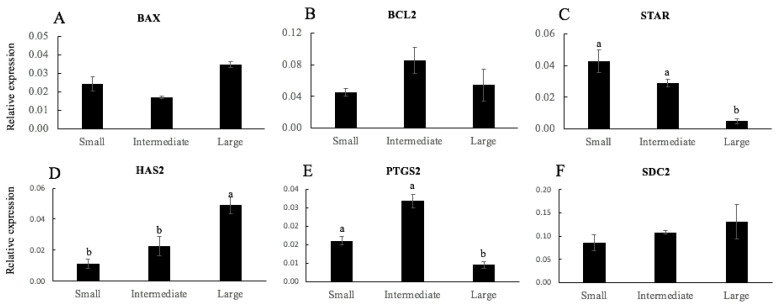
Relative gene expression of mRNA of *BAX* (**A**), *BCL2* (**B**), *STAR* (**C**), *HAS2* (**D**), *PTGS2* (**E**), and *SDC2* (**F**) of cumulus cells from sheep oocytes according to a clustering analysis of the total diameter (small, intermediate, and large). Different letters denote significant differences (*p* < 0.05, at least).

**Figure 4:**
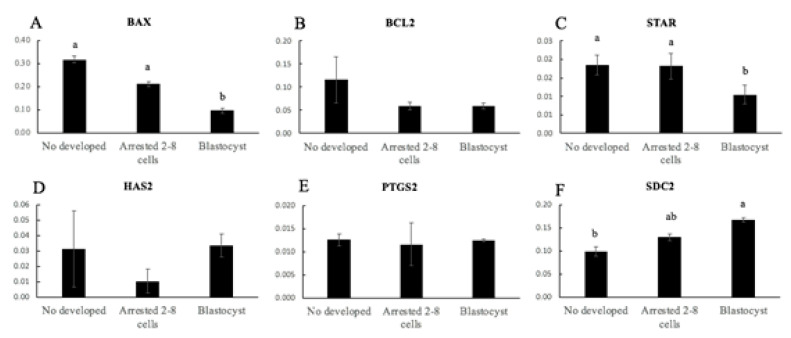
Relative gene expression of mRNA of *BAX* (**A**), *BCL2* (**B**), *STAR* (**C**), *HAS2* (**D**), *PTGS2* (**E**), and *SDC2* (**F**) and of cumulus cells from sheep oocytes according to the developmental stage reached (undeveloped, embryos arrested at the 2–8-cells stage, and blastocysts). Different letters denote significant differences (*p* < 0.05, at least).

The mRNA transcript relative abundance pattern of apoptosis genes (*BAX* and *BCL2*) and acquisition of oocyte competence (*STAR*, *HAS2*, *PTGS2*, and *SDC2*) were measured in CCs from oocytes classified according to a clustering analysis by total diameter (small, intermediate, or large) (Figure 3) and oocytes classified according to the developmental stage reached (not developed, embryos arrested at 2–8-cell stage, and blastocysts) (Figure 4). As shown in Figure 3, there were no differences in the relative mRNA transcript levels of apoptosis genes (*BAX* and *BCL2*) (Figure 3A,B, respectively) and *SDC2* (Figure 3F) between the groups. In large oocytes, the relative mRNA gene expression of *HAS2* (Figure 3D) was higher compared to small and intermediate oocytes (*p* < 0.05). However, the relative mRNA levels of *STAR* (Figure 3C) and *PTGS2* (Figure 3E) were lower in large oocytes when compared to small and intermediate oocytes (*p* < 0.05). 

In CCs from oocytes classified according to the developmental stage reached, there were no differences in the relative mRNA transcripts levels of *BCL2* (Figure 4B), *HAS2* (Figure 4D), and *PTGS2* (Figure 4E) between the groups. In the blastocyst group, the relative mRNA gene expression of *BAX* (Figure 4A) and *STAR* (Figure 4C) was lower than in the undeveloped group and in embryos arrested at the 2–8-cell stage (*p* < 0.02). In addition, the relative mRNA abundance expression of *SDC2* (Figure 4F) was lower in the undeveloped and blastocyst groups (*p* < 0.05). 

## 4. Discussion

Oocyte quality is crucial for the subsequent embryo development; therefore, the priority is the determination of individual biomarkers to select competent oocytes. In this context, the present work was conducted to improve IVP outcome in ovine by determining biomarkers of oocyte quality. To our knowledge, this research is the first to evaluate the morphometric parameters of oocytes and the relative gene expression of CCs as biomarkers by individual IVP in ovine. Although previous studies have reported that in vitro culture is more successful when the embryos are in large groups, due to reciprocal stimulation [27], some authors have shown that embryos produced in vitro individually had blastocyst rates similar to those cultured in groups [28], and the individual culture procedure had no effect on the embryo quality [29]. The present study utilized individual IVM, IVF, and IVC, which allowed for the monitoring of a single oocyte from the moment of maturation until the blastocyst stage. In addition, the individual IVP system allowed for the measurement of oocytes and relating their morphometric characteristics to their ability to produce embryos or not. We classified oocyte size into three categories, small, intermediate, and large, and a better outcome was obtained with the use of oocytes with 127.5 µm total diameter or 104.4 µm oocyte diameter (intermediate and large). Oocyte diameter as a criterion for oocyte selection has been extensively investigated in different species. In bovine species, oocytes acquired full meiotic competence at a diameter of 115 µm, and full developmental competence at a diameter of 120 µm [12,14]. In buffalo, developmental competence of oocytes may be achieved at a diameter of 145 μm [13]. In goats, higher rates of in vitro maturation were obtained in oocytes greater than 120 µm [15]. The difference in mean diameter of ovine oocytes in different studies may be due to different species or even different methodologies of oocyte diameter measurement. 

In the present study, according to total diameter, we observed that 20%, 55%, and 25% of the oocytes were classified as small, intermediate, and large, respectively. Although about 50% of small oocytes were capable of cleavage, only 3% were able to produce blastocysts (Table 4). However, intermediate and large oocytes yielded greater percentages of cleavage (80% and 90%, respectively) and were capable of producing 30–54% of blastocysts, respectively. 

Additionally, ZP thickness was measured to determine whether the ZP thickness could also be an indicator of oocyte quality. Interestingly, ZP thickness had a stronger correlation with in vitro embryo production, as oocytes with ZP ≥ 18.8 µm were able to obtain 100% cleavage and almost 68% blastocyst formation (Table 6). There are controversial reports regarding the effect of ZP thickness on oocyte quality. A previous study reported a negative correlation between ZP thickness and the ability of oocytes to resume meiosis and progress through the different stages of chromatin configuration in goat oocytes from early and antral follicles [30]. Another study reported that oocytes with high developmental competence had a significantly thicker ZP in equine oocytes [31]. In addition, ZP thickness in humans was associated with optimal folliculogenesis and/or oocyte maturation as well as blastocyst formation, with a slightly thinner ZP corresponding to significantly lower rates of conception or blastocyst development [32,33]. Regarding our results, we report that there was a relationship between the size of the oocyte and the thickness of the ZP, which together had an effect on the quality of the oocyte and subsequent embryonic development.

Furthermore, a statistical association between the total diameter, oocyte diameter, and ZP thickness with the formation of blastocysts was verified (Figure 1). The results of our study confirmed the findings of previous studies that reported that oocyte competence tended to increase with increasing oocyte size [12,13,14,15]. Therefore, the use of total diameter, oocyte diameter, and/or ZP thickness measurement as a noninvasive biomarker of oocyte quality would eliminate the subpopulation of small oocytes with poor quality, thereby improving IVP outcomes. 

In addition, we investigated the mRNA expression patterns of six genes related to apoptosis and oocyte quality in oocytes categorized by total diameter. *BCL2* and *BAX* are anti- and pro-apoptotic proteins, respectively, that participate in the mitochondria-dependent apoptosis pathway. Moreover, Van Soom et al. [34] reported that the different balance in the expression of pro- and anti-apoptotic genes may be a crucial factor determining the quality of the oocyte. In this study, greater expression of *BAX* transcript was observed in small oocytes, while lesser expression of *BCL2* transcript was observed for intermediate and large oocytes. These results are similar to those previously described by Opiela et al. [35], who reported that bovine immature oocytes that were brilliant cresyl blue (BCB)-negative (poor quality) had a higher expression of *BAX*, while mature BCB+ (high quality) showed a lower expression of *BCL2*. Another study reported that the upregulation of apoptotic genes in vitrified bovine oocytes may be an early indicator of reduced developmental competence following vitrification [36]. Oocyte-secreted factors, *BMP15* and *GDF9*, play crucial roles in the regulation of follicular development and oocyte maturation. In humans, high levels of *GDF9* and *BMP15* mRNAs in CCs are closely associated with oocyte competence and embryo quality [37]. In the same way, in goats, low levels of *GDF*-9 were expressed in immature oocytes at increasing levels at the time of cumulus cell expansion, after 12 h of IVM. On the contrary, in porcine oocytes, *GDF9* mRNA was highly expressed during the IVM of COCs and slowly decreased during the IVM process. In addition, canine oocytes expressed high levels of *GDF*-9 in oocytes at the earliest state of development but decreased during the maturation process until oocyte competence was reached [38]. Similar results were obtained in our investigation; the mRNA expression of *GDF9* was lower in oocytes with a greater capacity for embryonic development. The different results may be due to the maturation stage of the oocytes analyzed and/or the difference between species. In relation to cumulus expansion, gene expression of *GJA1* or connexin 43 was lower in large oocytes. *GJA1* has been proposed as a major mediator of cell–cell communication via gap junctions [39], being identified as a biomarker for the fertilization potential of human oocytes [40,41]. The reduced expression of *GJA1* in CCs after oocyte maturation was beneficial [42] because it was related to cumulus expansion and the decreased diffusion of cAMP and cGMP from the CCs to the oocyte, which occur in the final process of oocyte maturation [43].

The expression profile of six selected genes was examined in CCs from oocytes classified according to the total diameter or embryo stage reached (Experiment 2). The selected genes (*BAX*, *BCL2*, *STAR*, *HAS2*, *PTGS2*, and *SDC2*) were representative of previously identified CCs markers of oocyte quality and development. Several studies have demonstrated that the increased incidence of CCs’ apoptosis was negatively correlated to oocyte maturation and quality, fertilization, embryo development, and pregnancy outcomes [44,45,46]. Cumulus cells’ apoptosis in oocytes that reached the blastocyst stage was low, and this has been reported to have negative effects on oocyte quality and subsequent embryo development [44,45,46]. Furthermore, *BAX* expression was correlated with a higher apoptosis level and lower oocyte developmental competence in vitro [47]. We observed that *BAX* mRNA expression was significantly lower in CCs associated with blastocyst production than those associated with embryos arrested at the 2–8-cell stage and undeveloped oocytes, while *BCL2* mRNA concentrations did not vary in CCs, demonstrating that more competent oocytes cause less incidence of apoptosis. *STAR* is a critical enzyme in the steroidogenic activity of the ovary and is expressed in granulosa and CCs after the LH surge [48]. *PTGS2* is involved in prostaglandin E2 synthesis and has been reported to induce cumulus expansion [49]. Although mRNA levels of *PTGS2* in CCs are positively correlated with oocyte competence in other species such as cows [19], pigs [50], and humans [20], *PTGS2* mRNA expression was significantly lower in CCs from in vivo-matured rabbit COCs, which exhibited better CCs expansion and higher developmental competence after IVF [51]. The results from our study showed that the expression of *STAR* and *PTGS2* was lower in CCs of oocytes of high quality (from oocytes classified as large and oocytes that produced blastocysts). These findings are correlated with the lower expression for *STAR* and *PTGS2* that was associated with the further developmental potential from embryos to blastocysts in human cumulus cells [40]. On the other hand, the expression of *HAS2* and *SDC2* was high in CCs of oocytes of high quality (from oocytes classified as large and oocytes that produced blastocysts). *HAS2* are involved in final oocyte maturation [52]. Enhanced expression of *HAS2* in CCs has been reported to be associated with better oocyte and embryo development in humans and sheep [20,21]. The *SDC2* gene is involved in cell proliferation and cytoskeletal organization [47]. Previous studies associated the high quality of oocytes with cumulus expression of *SDC2*, which was reduced in poor-quality oocytes [47,53]. These results suggest that *BAX*, *STAR*, *HAS2*, *PTGS2*, and *SDC2* expression is strongly associated with oocyte quality and the ability of oocytes to produce blastocysts. 

## 5. Conclusions 

In conclusion, oocyte morphometric assessments (total diameter, oocyte diameter, and ZP thickness) may be used as noninvasive biomarkers of oocyte quality, being a more efficient and objective method to select high-quality oocytes compared to morphological criteria. Moreover, the relative gene expression in CCs of *BAX*, *STAR*, *HAS2*, *PTGS2*, and *SDC2* is also a good biomarker to select for high oocyte developmental competence, thereby improving IVP protocols in sheep. These findings could be applied in reproductive biotechnology to other species.

## Figures and Tables

**Table 1:** The gene-specific primers used for qPCR in oocytes and cumulus cells.

Target Gene	Gene Function	Primer Sequence (5′→3′)	Product Size	GenBank Accession No.
*PPIA*	Reference gene	F-TCAACCCCACCGTGTTCTTC R-GTCACCACCCTGGCACATAA	194	NM_001308578.1
*BAX*	Apoptosis	F-GTTGTCGCCCTTTTCTACTTTGC R-CAGCCCATGATGGTCCTGATC	89	NM_173894.1
*BCL2*	Apoptosis	F-GGAGCTGGTGGTTGACTTTC R-CTAGGTGGTCATTCAGGTAAG	518	NM_001077486.2
*BMP15*	Oocyte maturation	F-CTACGACTCCGCTTCGTGTGT R-AGTGCCATGCCACCAGAAC	69	NM_001031752.1
*GDF9*	Oocyte maturation	F-GAAGTGGGACAACTGGATTGTG R-CCCTGGGACAGTCCCCTTTA	71	NM_174681.2
*GJA3*	Gap junctions	F-TGCCTTTCGTTGTAACACTCA R-AGAACACATGAGCCAGGTACA	143	NM_174068.2
*HAS2*	Oocyte competence	F-CCTCATCATCCAAAGCCTG R-ACATTTCCGCAAATAGTCTG	138	NM_174079.2
*IGF2R*	Growth factor	FGCTGCGGTGTGCCAAGTGAAAAAG R-AGCCCCTCTGCCGTTGTTACCT	201	NM_174352.2
*STAR*	Oocyte competence	F-GCCAGGTGTTGAAGGCCA R-TCTTTAACAGACTTGGAGGCTTCC	104	NM_001009243.1
*PTGS2*	Oocyte competence	F-AGGAGGTCTTTGGTCTGGTG R-TCTGGAACAACTGCTCATCG	126	NM_001009432.1

**Table 2:** Mean values of individual and group (control) IVC parameters.

	Total	Maturation %	Fertilization Efficiency %	Cleavage %	Blastocyst %	Number of Cells/Blastocysts
Individual	199	81.0 ± 5.2	56.4 ± 1.2	78.2 ± 6.7	29.9 ± 1.3	108.9 ± 3.5
Control	167	81.9 ± 7.1	52.5 ± 3.1	77.9 ± 6.9	30.5 ± 1.3	88.9 ± 16.2

Data are expressed as mean ± SEM.

**Table 3:** Mean values of morphometric parameters of oocyte size in each generated cluster for total diameter, oocyte diameter, and ZP thickness.

Cluster	Total Diameter µm	Oocyte Diameter µm	ZP Thickness µm
1 (small)	88.4	78.3	9.7
2 (intermediate)	127.5	104.4	14.9
3 (large)	141.9	116.8	18.8

**Table 4:** Mean values of different developmental stages by clustering of morphometric parameters of oocyte by total diameter.

Cluster Total Diameter	Developmental Stages			
	Undeveloped %	Total Embryos at 2–8-Cell Stage %	Embryos Arrested at 2–8-Cell Stage %	Blastocysts %
1	54.8 ± 11.4 ^a^	45.1 ± 11.4 ^a^	42.0 ± 8.9	3.1 ± 3.1 ^a^
(small)	(13/24)	(11/24)	(10/24)	(1/24)
2	20.9 ± 9.3 ^b^	79.0 ± 9.3 ^b^	49.6 ± 5.3	29.4 ± 5.2 ^b^
(intermediate)	(12/76)	(64/76)	(40/76)	(24/76)
3	4.5 ± 4.5 ^b^	95.4 ± 4.5 ^b^	41.2 ± 13.3	54.2 ± 13.5 ^b^
(arge)	(2/33)	(31/33)	(12/33)	(19/33)

Data are expressed as mean ± SEM. Values within columns are significantly different (^a,b^ *p* < 0.05).

**Table 5:** Mean values of different developmental stages by clustering of morphometric parameters of oocytes by oocyte diameter.

Cluster Oocyte Diameter	Developmental Stages			
	Undeveloped %	Total Embryos at 2–8-Cell Stage %	Embryos Arrested at 2–8-Cell Stage %	Blastocysts %
1	50.8 ± 11.0 ^a^	49.2 ± 11.0 ^a^	35.0 ± 1.6	14.1 ± 9.4 ^a^
(small)	(12/28)	(16/28)	(10/28)	(6/28)
2	17.2 ± 7.8 ^b^	82.8 ± 7.8 ^b^	52.3 ± 10.1	30.5 ± 10.1 ^ab^
(intermediate)	(9/71)	(62/71)	(41/71)	(21/71)
3	17.1 ± 5.7 ^b^	82.8 ± 5.7 ^b^	38.4 ± 9.6	44.4 ± 3.9 ^b^
(large)	(7/34)	(27/34)	(11/34)	(16/34)

Data are expressed as mean ± SEM. Values within columns are significantly different (^a,b^ *p* < 0.05).

**Table 6:** Mean values of different developmental stages by clustering of morphometric parameters of oocytes by ZP thickness.

Cluster ZP Thickness	Developmental Stages			
	Undeveloped %	Total Embryos at 2–8-Cell Stage %	Embryos Arrested at 2–8-Cell Stage %	Blastocysts %
1	41.8 ± 10.3 ^a^	58.2 ± 10.3 ^a^	31.5 ± 12.4	26.7 ± 3.9 ^a^
(small)	(24/60)	(45/60)	(21/60)	(15/60)
2	6.6 ± 4.1 ^b^	93.3 ± 4.1 ^b^	64.7 ± 6.3	28.6 ± 9.6 ^a^
(intermediate)	(3/56)	(53/56)	(34/56)	(19/56)
3	0.0 ± 0.0 ^b^	100 ± 0.0 ^b^	32.4 ± 12.4	67.6 ± 12.4 ^b^
(large)	(0/17)	(17/17)	(6/17)	(11/17)

Data are expressed as mean ± SEM. Values within columns are significantly different (^a,b^ *p* < 0.05).

## Data Availability

Data are available from the corresponding upon request.
